# Current Status of Norovirus Infections in Children in Sub-Saharan Africa

**DOI:** 10.1155/2015/309648

**Published:** 2015-11-16

**Authors:** Samuel Munalula Munjita

**Affiliations:** Department of Biomedical Sciences, School of Medicine, University of Zambia, 15101 Lusaka, Zambia

## Abstract

Noroviruses are a leading cause of acute sporadic gastroenteritis worldwide. In Sub-Saharan Africa, information regarding norovirus infections in children is scarce. A systematic review of studies performed between 1993 and June 2015 was conducted to establish the genotypic distribution and prevalence of norovirus infections in children (≤17) in Sub-Saharan Africa. Analysis of data from 19 studies involving 8,399 samples from children with symptomatic and nonsymptomatic gastroenteritis revealed prevalence of 12.6% (range 4.6% to 32.4%). The prevalence of norovirus infections was higher in symptomatic children (14.2%) than asymptomatic children (9.2%). Genogroup II (GII) was the most prevalent genogroup accounting for 76.4% of all the reported norovirus infections. The rest of the infections were GI (21.7%) and GI/GII (1.9%). The most common genotypes were GII.4 (65.2%), GI.7 (33.3%), and GI.3 (21.3%). These statistics were calculated from studies carried out in 12 out of 48 Sub-Saharan African countries. Therefore, more studies involving several countries are required to determine fully the epidemiology of noroviruses and their contribution to childhood diarrhoea in Sub-Saharan Africa.

## 1. Introduction

Acute gastroenteritis is a common illness which is debilitating and life-threatening in children younger than five years of age [1]. Globally, diarrhoeal diseases claim the lives of 800,000 children under the age of five years every year [2]. Most of the deaths occur in Sub-Saharan Africa and south Asia [3]. Noroviruses account for 12% of all cases of sporadic acute gastroenteritis in children under the age of five years worldwide and approximately 200,000 children die from norovirus associated gastroenteritis in developing countries [4, 5]. Noroviruses are small, single-stranded, positive-sense, RNA viruses belonging to the family* Caliciviridae* and the genus* Norovirus* [5]. They are divided into six genogroups and over thirty-one genotypes [5]. The epidemiology of these viruses is well described in developed countries. However, the exact epidemiology and contribution of noroviruses to acute gastroenteritis in children in Sub-Saharan Africa remain largely unclear. Therefore, a systematic scientific literature review of studies in Africa was carried out to establish the genotypic distribution and prevalence of symptomatic and nonsymptomatic norovirus infections in children in Sub-Saharan Africa.

### 1.1. Genome Organisation and Genetic Diversity of Noroviruses

Noroviruses have a linear and polyadenylated genome surrounded by nonenveloped, icosahedral capsid of 27 to 40 nm in diameter [5]. The genome of noroviruses that infect humans is organised into three open reading frames (ORFs). ORF1 is the largest and encodes a polyprotein precursor for the seven nonstructural proteins [5, 6]. ORF2 and ORF3 encode the major and minor structural capsid proteins VP1 and VP2, respectively [6]. Murine norovirus genomes comprise an additional ORF4, which encodes virulence factor I [7]. The genus,* Norovirus*, is divided into six genogroups (GI–VI) based on the amino acid sequence of the major structural protein VP1 [5, 8]. Human pathogens are found in GI, GII, and rarely GIV. There is extensive genetic diversity among norovirus strains and new variants emerge almost every 2-3 years [5, 6]. GI is divided into 9 genotypes and GII into 22 genotypes [5]. The (human) Norwalk virus is the prototype of the genus and is designated as GI.1. GIII comprises bovine and ovine strains and GV murine noroviruses [5]. Swine and canine strains are classified into GII and GIV or GVI, respectively [5]. In humans, GII.4 noroviruses predominantly cause the vast majority of gastroenteritis outbreaks worldwide [9, 10].

### 1.2. Transmission and Clinical Characteristics of Norovirus Infections

Acute norovirus infection is characterised by nonspecific symptoms such as vomiting, nausea, abdominal cramps, myalgias, and intense watery nonbloody diarrhoea that commonly resolves in 2-3 days [5]. Children and immunocompromised individuals may experience a more prolonged and severe disease course lasting for a few weeks to years [11, 12]. Sequelae of prolonged norovirus associated gastroenteritis may include irritable bowel syndrome, necrotising enterocolitis, convulsions, and encephalopathy [13–15]. Transmission of noroviruses occurs through the faecal-oral route either by direct contact with infected individuals or via contaminated surfaces, food, water, and aerosolised particles from infected vomitus or stool [5]. Outbreak control and prevention strategies are limited to the use of disinfectants and hand sanitisers.

## 2. Methods

### 2.1. Search Strategy and Selection Criteria

A systematic scientific literature review of norovirus studies in Sub-Saharan Africa published in peer reviewed journals in electronic databases such as PubMed, Medline, Scopus, and Google Scholar from 1993 to June 2015 was performed using keywords such as “gastroenteritis”, “children”, “norovirus”, “Africa”, and “name of country” alone and in various combinations. To avoid leaving out any studies that were not available in major scientific databases or nonindexed journals, Google search was also used. Master of Science theses whose contents have not been published in any journal were also included in the review. Studies outside Sub-Saharan Africa and those involving adult participants alone were excluded. For studies involving both children and adults, only data from children was extracted and included in this review. All studies describing norovirus infections in symptomatic and asymptomatic children were included. Due to the scarcity of information on norovirus infections in children in Sub-Saharan Africa, studies of all durations (≥1 month) were included in this systematic review.

### 2.2. Data Extraction and Analysis

When reviewing the studies, frequencies of genogroup I and of genogroup II genotypes were calculated independently. Classification of genotypes into polymerase and capsid genotypes was not considered in the analysis due to lack of information. To calculate the approximate prevalence of norovirus infections in Sub-Saharan Africa, only reverse transcription PCR based studies were included. However, a reverse transcription PCR based study by Wolfaardt et al. that used calicivirus positive samples only to calculate the prevalence of Norwalk virus in South Africa was excluded from the calculation [16]. In studies, where the overall prevalence of norovirus infections was not included, prevalence was calculated using the information extracted from the articles. Seasonality of norovirus infections was accepted and included in this review only when the study duration was ≥12 months.

## 3. Results

Thirty-five studies were identified in Africa and only 23 were in Sub-Saharan Africa [17–39]. Nineteen studies had sufficient data to determine the total prevalence of norovirus infections but only seventeen studies had sufficient data to allow the determination of the prevalence of genogroups I and II [17–34, 36]. Relative frequencies of norovirus GI and GII genotypes were determined from 11 studies only due to lack of data from the other studies [17, 19, 20, 22–25, 29, 31, 33, 36]. Only three studies had some information on the HIV status of study participants [18, 22, 33]. The duration of most studies was less than 12 months. Consequently, the seasonality of norovirus infections could not be established in many countries. Four studies reported the presence of norovirus recombinants [23, 28, 31, 36] and one study investigated the role of genetic factors on the susceptibility to norovirus infections [29].

### 3.1. Prevalence of Norovirus Infections

**Figure 1:**
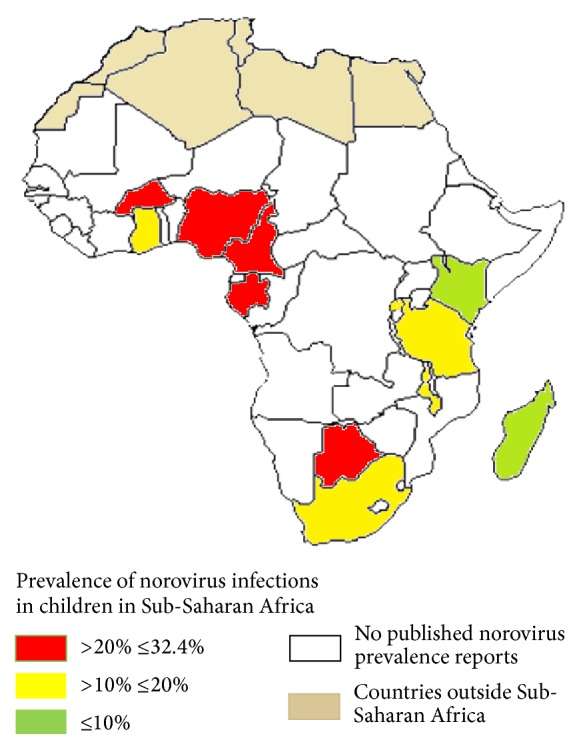
Countries where norovirus infections in children have been reported in Sub-Saharan Africa. Countries with high norovirus prevalence (>20% ≤32.4%) are represented by the red colour. Yellow: countries with norovirus prevalence between 10% and 20%. Light green: countries with less than 10% norovirus prevalence. At the time of writing this review, there were no published reports about prevalence rates of norovirus infections in* children* in other Sub-Saharan African countries apart from the ones represented by red, yellow, and light green colours.

In Sub-Saharan Africa, norovirus associated gastroenteritis outbreaks were first reported in South Africa in 1993 [37]. Hawaii (GII.1) and Norwalk (GI.1) strains were identified as causative agents during the two successive outbreaks. Subsequent seroepidemiological studies in South Africa and in Southern Africa (South Africa, Angola, Zimbabwe, Mozambique, and Namibia) published in 1996 and 1999 reported higher prevalence of IgG antibodies against the prototype Norwalk virus (GI.I), 55.5% and 94.4% seropositivity, respectively [38, 39]. Another study recorded 3% prevalence of Norwalk virus in human calicivirus (HuCVs) positive stool specimens obtained between October 1991 and October 1995 from South African patients with sporadic gastroenteritis [16]. Analysis of results from 19 reverse transcriptase-PCR based studies involving 8,399 samples from children (≤17 years) with symptomatic and nonsymptomatic gastroenteritis revealed 12.6% (1,057/8,399) prevalence of norovirus infections in Sub-Saharan Africa [18–34, 36]. The prevalence ranged from 4.6% to 32.4% (Table 1, Figure 1). Norovirus infections were more prevalent in children ≤5 years [18–34, 36]. The prevalence of norovirus infections was higher in symptomatic children (14.2%, 807/5680) than asymptomatic children (9.2%, 250/2719). However, higher prevalence of norovirus infections in asymptomatic children was reported in Botswana (31%), Burkina Faso (24.8%), and Cameroon (29.6%) [17, 18, 28].

### 3.2. Mixed and Single Virus Infections in Norovirus Positive Diarrhoea Samples

Four studies, from South Africa, Nigeria, Malawi, and Gabon, clearly reported the occurrence of noroviruses in diarrhoea samples as single or mixed infections [20, 26, 33, 34]. In the study from South Africa, 77% (27/35) of the norovirus infections occurred as single virus infections whereas 23% (8/35) of the norovirus positive specimens were coinfected with rotaviruses, adenoviruses, sapoviruses, and astroviruses [33]. In Ife, Nigeria, norovirus single infections were found in 64.3% (9/14) of the norovirus positive diarrhoea samples. The rest of the norovirus positive specimens were coinfected with rotaviruses [26]. In Malawi, 1.5% of the norovirus positive diarrhoea samples were coinfected with rotaviruses [20]. A study in Gabon had 41.1% of norovirus infections occurring as single infections; 58.9% of the norovirus diarrhoea samples were coinfected with rotaviruses, adenoviruses, sapoviruses, and astroviruses [34]. Studies from Tanzania, Northern region of Ghana, and Zanzibar indicated the presence of mixed infections but fell short of mentioning the percentage of norovirus positive diarrhoea samples that had single or mixed infections [21, 25, 35]. The rest of the studies included in this review had no information about the occurrence of single or mixed infections in norovirus positive samples.

### 3.3. Distribution of Genogroups and Genotypes

**Figure 2:**
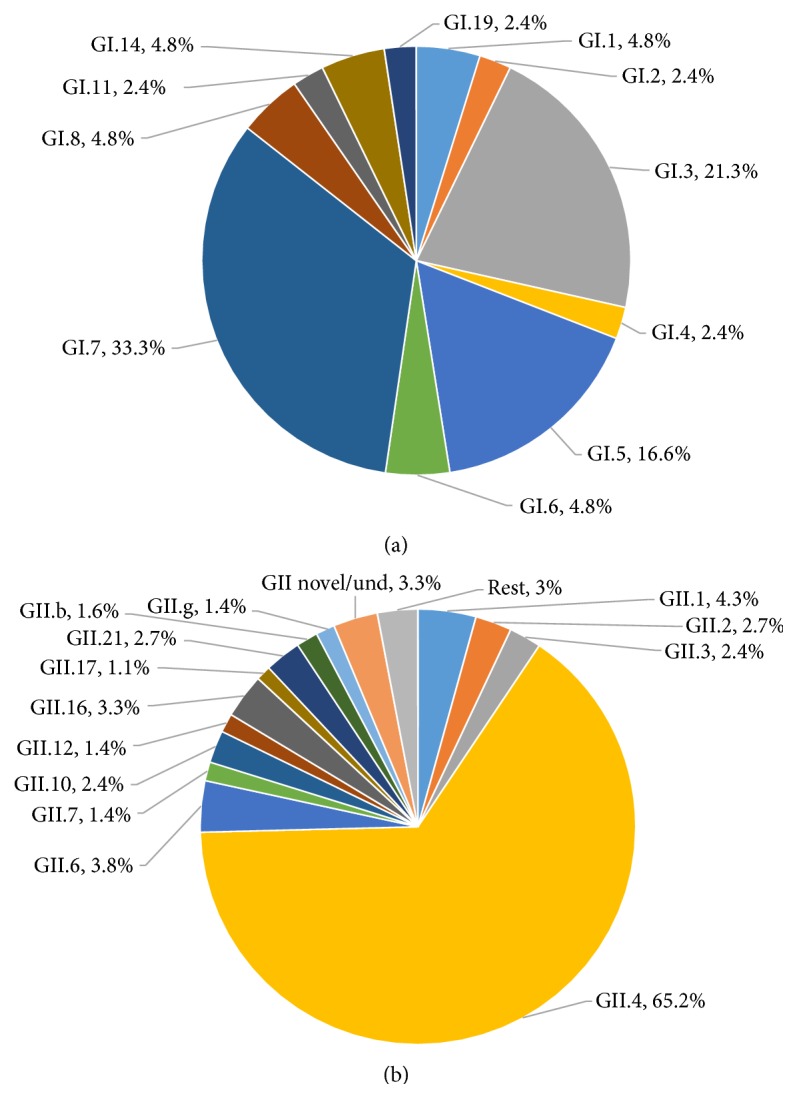
Distribution of GI and GII norovirus genotypes (capsid and polymerase combined) in Sub-Saharan Africa. (a) Relative frequencies of GI genotypes among a total of 42 GI norovirus positive samples from nine studies. GI.7 and GI.3 norovirus infections were more common than any other GI genotype. (b) Relative frequencies of GII genotypes among a total of 368 GII norovirus positive samples from nine studies. GII.4 (65.2%) was the most prevalent genotype. Und: undefined. Rest: GII.e, GII.8, GII.11, GII.13, GII.14, and GII.15.

GII was the most common genogroup accounting for 76.4% (730/955) of all the reported norovirus infections from 17 studies in Sub-Saharan Africa. Two studies had no information on the genogroups and one study from Nigeria (included in the 17 studies) did not sequence all the norovirus positive samples to determine the genogroups [21, 27, 30]. The rest of the infections were GI (21.7%, 207/955) and GI/GII (1.9%, 18/955). Analysis of nine studies that had information on the prevalence of GI and GII genotypes revealed a great diversity of norovirus genotypes in Sub-Saharan Africa [17, 20, 22, 23, 25, 29, 31, 33, 36]. Figures 2(a) and 2(b) show the relative frequencies of norovirus genotypes in Sub-Saharan Africa. GII.4 (65.2%) was the most prevalent GII genotype associated with acute sporadic gastroenteritis. GI.7 (33.3%) and GI.3 (21.3%) were the most common GI genotypes.

### 3.4. Distribution of Norovirus Recombinants

Norovirus recombinants were only reported in four studies in Burkina Faso, Madagascar, Ghana, and South Africa [23, 28, 31, 36]. The reported recombinants included GII-8/GII-14 in Ghana in 2006, GII.7/GII.6 in Burkina Faso in 2013, and a potential recombinant (closer to Hu/NoV/GII.1/Hawaii/1971/US) in Madagascar in 2007 [23, 28, 31]. The most comprehensive study thus far to have reported the presence of several GII intergenotype and two intragenotype GII.4 recombinants was carried out by Mans et al., in South Africa [36]. Three of the combinations represented novel recombinants, namely, GII.P not assigned (NA)/GII.3, GII.P4 New Orleans 2009/GII.4 NA and GII.P16/GII.17 [36]. Other recombinants included GII.P21/GII.2, GII.P21/GII.3, GII.Pe/GII.4 Sydney 2012, GII.Pg/GII.12, GII.Pg/GII.1, and GII.Pe/GII.4 Osaka 2007, GII.P4 New Orleans 2009/GII.4 Sydney 2012, and GII.P7/GII.6 [36].

### 3.5. Genetic Susceptibility to Norovirus Infections

Norovirus infections were less prevalent in Burkinabe children with blood group A (Odds Ratio 0.31; *P* = 0.054) or secretor-negative phenotype (Odds Ratio 0.18; *P* = 0.012) [29]. Lewis-negative children were more susceptible to GII but were not infected with any GI noroviruses. Children with blood group B were infected by GII.4 strains whereas secretor-positive children with blood group O were infected with a wide variety of genotypes [29].

### 3.6. Norovirus Gastroenteritis in HIV Positive Children

Three smaller studies in Tanzania, South Africa, and Cameroon reported the prevalence of norovirus infections in HIV positive people [18, 22, 33]. The study in Cameroon involved HIV positive adults only and thus it was excluded from the analysis. In Tanzania, Moyo et al. found prevalence of 21.2% in HIV positive children compared to 10.3% in HIV negative children [22]. In South Africa, a study carried out by Mans et al. reported that 42.8% (15/35) of children with norovirus gastroenteritis were infected with HIV [33]. Eight out of fifteen HIV infected people had chronic diarrhoea.

### 3.7. Seasonality of Norovirus Gastroenteritis in Sub-Saharan Africa

The seasonal distribution of noroviruses varied between different Sub-Saharan African countries. In South Africa and Madagascar, norovirus detection peaked in November and December 2008 while Malawi had two peak norovirus seasons, August to November and February to March between 1997 and 2007 [20, 31, 33]. Peak detection in Tanzania occurred in April [22]. Ghana and Nigeria experienced high norovirus detection during the dry season, from October to May and October to January, respectively [23, 24, 26, 27].

## 4. Discussion

### 4.1. Prevalence of Norovirus Infections

Data from this review shows that the prevalence (12.6%) of norovirus infections in children in Sub-Saharan Africa is higher than previously thought. In most of the studies included in this review, the prevalence of norovirus was higher in the cases than in the controls [20, 22, 27, 32, 35]. Some studies in Botswana, Burkina Faso, and Cameroon reported higher prevalence in asymptomatic children [17, 18, 28]. The reasons for this phenomenon seen in these countries are unknown. It can only be speculated that the findings from such studies were a true reflection of the situation or some of the individuals included in these studies did not have truly asymptomatic infections or their parents/guardians had incomplete recall of diarrhoeal symptoms. Persistent shedding of the virus from resolved acute norovirus diarrhoeal infections could also have contributed to these events. On the other hand, the samples sizes for the studies conducted in Botswana (100 children) and Cameroon (54 children) were too small to draw serious conclusions [17, 18]. Moreover, the study in Cameroon only involved asymptomatic children and thus it is difficult to really know the extent to which norovirus infections contribute to diarrhoea in Cameroonian children.

Generally, most of the studies included in this review had smaller sample sizes. Only three studies had sample sizes above 1000 [19, 20, 22]. Therefore, norovirus prevalence observed in smaller studies may be enlightening but not conclusive. Longitudinal studies with large sample sizes are required to evaluate the exact norovirus prevalence and its contribution to diarrhoeal disease in children in Sub-Saharan Africa. The calculated 12.6% prevalence of norovirus infections came from studies in 12/48 Sub-Saharan African countries. There was no information about norovirus infections from the rest of the 35 countries. Therefore, this is not a representative prevalence rate. More studies in all the countries are required in order to come up with a representative figure.

Additionally, the duration of many of the studies was less than 12 months, and hence results from such studies are but just an eye-opener of the role of noroviruses in childhood diarrhoea in those countries [18, 19, 21, 25–29, 31, 35]. Consequently, the seasonality of norovirus infections in the country where the studies were carried out could not be established. Knowing the season within which norovirus infections frequently occur may help with proper planning for outbreaks and efficient utilisation of health resources.

### 4.2. How Much Diarrhoea Is Caused by Noroviruses in Sub-Saharan Africa?

Although the prevalence of norovirus infections is approximately 12.6% in Sub-Saharan Africa, the contribution of noroviruses to acute and chronic diarrhoea in this subregion is still obscure.

Detection of norovirus in a stool sample from a child with gastroenteritis does not always mean that this is the cause; there may be mixed infections with other bacterial and viral agents. As true as this statement may sound, many studies included in this review did not investigate the occurrence of noroviruses as single or mixed virus infections in norovirus positive diarrhoea samples. For studies, such as those conducted in South Africa, Nigeria, and Malawi, that investigated occurrence of noroviruses as single or mixed infections with other viral pathogens, they did not investigate coinfections with protozoal and bacterial agents [20, 26, 33, 34]. Therefore, it is still largely difficult to pinpoint the exact share of diarrhoea burden caused by noroviruses in Sub-Saharan Africa.

### 4.3. Norovirus Genotypes in Sub-Saharan Africa

Out of a total of 19 studies that were included in this review, only 9 had information of the prevalence of GI and GII genotypes, representing a total of 368 GII genotypes and 42 GI genotypes. This, coupled with smaller sample sizes and fewer studies, makes the distribution of norovirus genotypes highlighted in this review unrepresentative of actual norovirus genotypes that may be circulating in Sub-Saharan Africa [17, 20, 22, 23, 25, 29, 31, 33, 36]. Most of the studies ended their research on determining the genogroups of noroviruses. However, among the genotyped samples, GII.4, GI.3, and GI.7 were most associated with diarrhoea. Results from this systematic review indicate that the exact contribution of different norovirus genotypes to diarrhoea in children in Sub-Saharan Africa is far from being established.

### 4.4. Norovirus Gastroenteritis in HIV Positive Children

Noroviruses have been shown to cause chronic and persistent gastroenteritis in immunocompromised individuals such as HIV/AIDs patients, lasting several weeks to years [11, 12]. By the end of 2013, there were approximately 35 million people living with HIV worldwide [40]. Sub-Saharan Africa accounted for 71% (approximately 24.7 million people) of the global HIV infections. With such a huge percentage of people infected with HIV, noroviruses may be silently causing undocumented severe and prolonged gastroenteritis in Sub-Saharan Africa. In this review, only two studies reported the prevalence of norovirus infections in HIV positive children [22, 33]. In South Africa, more than 50% (8/15) of HIV positive children had chronic norovirus diarrhoea. The results were in agreement with the findings from across the globe that noroviruses tend to cause persistent diarrhoea in immunocompromised individuals. Although the results from these two studies may not be adequate to draw conclusions, they point to the potential devastating role of HIV in chronic norovirus gastroenteritis. Large scale research in Sub-Saharan Africa is required to correctly understand the impact of HIV/AIDS in norovirus associated gastroenteritis.

### 4.5. Risk Factors and Transmission Dynamics of Noroviruses

Whilst all the studies included in this systematic review provided evidence on the prevalence of norovirus infections in children in Sub-Saharan Africa, none of them critically looked at the possible risk factors and transmission dynamics of noroviruses. It would be worthwhile to investigate the transmission dynamics and risk factors associated with norovirus infections in Sub-Saharan Africa.

## 5. Conclusion

The prevalence of norovirus infections in children with symptomatic and nonsymptomatic gastroenteritis in Sub-Saharan Africa is approximately 12.6%. However, most of the studies from which this figure was derived had small sample sizes and were of a shorter duration. It is therefore prudent that the epidemiology of noroviruses and their contribution to childhood diarrhoea and deaths in Sub-Saharan Africa should be investigated further using large scale longitudinal studies.

## Figures and Tables

**Table 1:** Norovirus infections in children in Sub-Saharan Africa.

Country	Prevalence of norovirus (%)				Sample size	Year of samples collected	Age (years)	Reference
	Total	GI	GII	GI/GII				
Botswana	24	3	21	0	100	2000–2006	≤1–≥3	[17]
Cameroon	29.6	12.9	16.7	0	54	Oct–Dec 2009	5–15	[18]
Cameroon	4.6	2.2	2.4	0	146 (1244 samples)	Sep 2011–Aug 2012	1–17	[19]
Malawi	11.3	1.8	9.4	0.1	1941	July 1997–June 2007	<5	[20]
Tanzania	13.7	—	—	—	270	Dec 2005–Feb 2006	<5	[21]
Tanzania	14.3	0.9	13.3	0.1	1266	2010-2011	≤2	[22]
Ghana	15.9	3.7	12.2	0	82	Aug 1998–July 2000	≤2	[23]
Ghana	16.4	1.3	15.1	0	152	Feb 2011–Feb 2012	≤5	[24]
Ghana	7.4	1.4	6	0	367	Nov 2005–Jan 2006	≤11	[25]
Nigeria	25.5	1.8	23.6	0	55	June 2010–Jan 2011	≤5	[26]
Nigeria^*∗*^	32.4	2.6	4.3	1.6	100	Nov 2007–Jan 2008	<5	[27]
Burkina Faso	22.2	8.8	10.5	2.9	418	Nov 2005–Jan 2007	≤10	[28]
Burkina Faso	12	2.3	9.7	—	309	May 2009–Mar 2010	<5	[29]
Kenya	6.3	—	—	—	206	Jan 2007–June 2010	<14	[30]
Madagascar	5.9	1.7	4.2	0	237	Nov 2005–Jan 2008	≤16	[31]
Rwanda	11	3.8	7.2	0	706	Nov 2009–June 2012	≤5	[32]
South Africa	14.3	1.2	12.7	0.4	245	Jan–Dec 2008	≤13	[33]
Gabon	23	9.1	13.9	0	317	Mar 2010–June 2011	<5	[34]
Tanzania (Zanzibar)	11.8	0.6	11.2	0	330	April–July, 2011	<5	[35]

^*∗*^Study did not sequence all the norovirus positive samples to determine the genogroups.
